# Short Snips: YouTube Shorts Recommendations for Hair Loss and Alopecia

**DOI:** 10.1111/jocd.16608

**Published:** 2024-10-03

**Authors:** Michelle Ko, David Kim, Emily Newsom, Carolyn Goh

**Affiliations:** ^1^ David Geffen School of Medicine University of California, Los Angeles California USA; ^2^ Division of Dermatology University of California, Los Angeles California USA

**Keywords:** alopecia, hair loss, social media, treatment, YouTube Shorts

**Abbreviation**
OTC: over‐the‐counter.

To the Editor

As 87.6% of participants watch health‐related content on YouTube, and 84.7% make decisions on the basis of what they watch, dermatologists should be aware of the information and trends on this platform [1]. Furthermore, for alopecia, YouTube trends have shown a linear increase worldwide over time for the search term “hair loss,” from 16 in 2008 to 94 in 2024 on a scale of 0–100 (0 referring to not enough data, 50 half as popular, and 100 peak popularity). Though many studies have characterized hair loss treatment trends on YouTube [2, 3, 4], none have done so with YouTube Shorts, a short‐form video platform created in 2021 to rival competitors such as TikTok and Instagram Reels. Here, we conducted a cross‐sectional analysis to assess treatment recommendations for alopecia or hair loss in YouTube Shorts.

On April 14, 2024, the following search phrase was searched in YouTube.com: “#shorts + treatment (alopecia OR hair loss).” The “relevance” filter was used, and the search was conducted in “incognito mode” for the web browser to reduce influence from personal search algorithms. The first 150 videos to appear were collected. Exclusion criteria included videos that were not in English, had < 1000 views, and were not relevant. For all included videos, an engagement score was calculated with the following equation: [(likes + comments)/views] × 100. Type of video speaker, type of hair loss mentioned, and treatment recommendations were recorded for each video. Videos were categorized into recommendations that (1) can be carried out by patients independently, (2) require medical assistance, and (3) are “home remedies.” This study was modeled after research by Nickles et al. [5].

Of 150 videos collected, 80 met the inclusion criteria. The highest percentage of videos were produced by patients/bloggers (36.3%), with the highest mean engagement score seen with videos produced by trichologists (2.9%), dermatologists (2.8%), and patient/bloggers (2.7%) (Table 1). The majority of videos did not specify which hair loss subtype they were aiming to treat, though 13.75% mentioned alopecia areata and 2.5% mentioned female/male pattern baldness. The majority of videos (56.25%) recommended treatments that could be carried out independently, such as with over‐the‐counter (OTC) topical medications and cosmetics, wigs and caps, and dermarollers. Thirty videos recommended treatments that required medical assistance, such as prescription topicals, oral medications such as baricitinib, platelet‐rich plasma (PRP), laser therapy, steroid injections, and hair transplants. Lastly, 11 videos recommended “home remedies,” such as drinks of cucumber, potato, and curry leaves, and topical mixtures of aloe, shea butter, and coconut oil. Leech therapy was also recommended by one video. The top recommendations for alopecia included minoxidil (26.3%, *n* = 21), alternative therapies (8.8%, *n* = 13), and OTC topical treatments (16.3%, *n* = 13) (Table 2). Of note, oral finasteride and topical minoxidil are the only US Food and Drug Administration (FDA)‐approved medications for androgenetic alopecia. JAK inhibitors baricitinib, ritlecitinib, and deuruxolitinib are the only FDA‐approved medications for severe alopecia areata and have a black box warning for serious infection, malignancy, and thrombosis [6]. Other treatments are routinely used off label.

**TABLE 1:** Descriptive characteristics of YouTube Shorts recommendations for hair loss/alopecia.

Video speaker	% (*n*)	Mean views	Mean likes	Mean comments	Mean engagement	For alopecia areata^c^, % (*n*)	For androgenetic alopecia, % (*n*)	No subtype specified, % (*n*)	Recommendations		
									Patients can carry out independently, % (*n*)	Require medical assistance, % (*n*)	Home remedies', % (*n*)
Patient/Blogger	36.3% (29)	1 017 486	29 842	328	2.7%	0% (0)	0% (0)	42.7% (29)	40.0% (18)	20.0% (6)	54.6% (6)
Group practice^b^	22.5% (18)	946 500	4212	122	1.1%	50% (5)	0% (0)	19.1% (13)	8.9% (4)	50.0% (15)	0% (0)
Dermatologist^a^	13.8% (11)	931 536	34 092	570	2.8%	20% (2)	100% (2)	10.3% (7)	15.6% (7)	13.3% (4)	9.1% (1)
Other^c^	12.5% (10)	793 000	20 345	465	2.2%	20% (2)	0% (0)	11.8% (8)	11.1% (5)	13.3% (4)	27.3% (3)
Hair product company^d^	7.5% (6)	1 432 667	38 183	498	1.7%	10% (1)	0% (0)	7.4% (5)	13.3% (6)	0% (0)	0% (0)
Trichologist	5.0% (4)	924 975	40 761	184	2.9%	0% (0)	0% (0)	5.9% (4)	6.7% (3)	0% (0)	9.1% (1)
Non‐dermatologist physician	2.5% (2)	307 000	6750	309	2.5%	0% (0)	0% (0)	2.9% (2)	4.4% (2)	3.3% (1)	0% (0)
Total	80	970 386	24 067	337	2.2%	10	2	68	45	30	0

^a^ Eight featured US‐based, board‐certified physicians. Three featured a non‐US dermatologist.

^b^ Channels self‐identified as skin, hair, and cosmetic dermatology group practices.

^c^ Other included miscellaneous video speakers included health (Ayurveda medicine, chiropractor, health info channels, WebMD), hair practitioner (barbers), and a video channel dedicated to trending videos.

^d^ Companies included “ThickFiber,” “Toppik,” and “1 Hair Stop.”

**TABLE 2:** Summary of the top 12 recommended hair loss/alopecia treatments in the YouTube Short videos analyzed.

Top 12 recommended treatments for hair loss/alopecia on YouTube Shorts	Percentage of videos recommending treatment (*n*, total videos = 80)	No. for alopecia areata (*n* = 10)	No. for androgenetic alopecia (*n* = 2)	No. for unspecified hair loss (*n* = 68)
Minoxidil	26.3% (*n* = 21)	1	2	18
Unspecified minoxidil	17.5% (*n* = 14)	1	0	13
Minoxidil 5%	8.8% (*n* = 7)	0	2	5
Alternative therapy^a^	16.3% (*n* = 13)	1	0	12
OTC topical	16.3% (*n* = 13)	1	0	12
PRP	13.8% (*n* = 11)	0	0	11
Finasteride	10.0% (*n* = 8)	0	0	8
Injection	10.0% (*n* = 8)	7	0	1
Hair transplant	8.8% (*n* = 7)	0	0	7
Dermaroller	7.5% (*n* = 6)	0	0	6
Hair care	3.8% (*n* = 3)	0	0	3
Laser	3.8% (*n* = 3)	0	1	2
OTC oral supplement	3.8% (*n* = 3)	0	0	3
Wig	3.8% (*n* = 3)	0	0	3

^a^ Alternative therapies include homemade topical therapies such as rosemary oil, castor oil, mustard oil, pumpkin seed oil, peppermint oil, coconut oil, saw palmetto, apple cider vinegar, onion juice, coffee, aloe, shea butter, and mixtures containing fenugreek seed and curry leaves. Other recommendations included drinks with cucumber, potato, curry leaves, and water, Ayurveda therapy, and leech therapy.

Though dermatologists created < 15% of the videos, they had high viewer engagement levels. By utilizing popular social media platforms such as YouTube Shorts, dermatologists have the opportunity to bring their expertise outside of the medical and clinical environment to share evidence‐based practices and information with a wider reach of audiences in the community. Dermatologists may use the platform to directly dispute misinformation, either by creating reaction videos to “myths” being circulated widely online or by creating videos that address such misinformation using the “Remix” option in YouTube [7]. Additionally, dermatologists may increase viewer engagement by posting consistently and providing informative content consistent with previous research [7], as well as considering the entertainment value of their videos.

## Ethics Statement

Reviewed and approved for IRB exemption by the Institutional Review Board at the University of California Los Angeles, exemption #IRB#24‐000011.

## Conflicts of Interest

C.G. has the following financial disclosures: Pfizer, Inc.—Speaker and Consultant—Fees; BMJ BestPractices—Honoraria; Pelage Pharmaceuticals—Advisory Board—Fees. All other authors do not have any proprietary interests or conflicts of interest related to this submission to disclose.

## Data Availability

Research data are not shared.
